# Functional Contributions of Positive Charges in the Pore-Lining Helix 3 of the *Bordetella pertussis* CyaA-Hemolysin to Hemolytic Activity and Ion-Channel Opening

**DOI:** 10.3390/toxins9030109

**Published:** 2017-03-16

**Authors:** Chattip Kurehong, Chalermpol Kanchanawarin, Busaba Powthongchin, Panchika Prangkio, Gerd Katzenmeier, Chanan Angsuthanasombat

**Affiliations:** 1 Bacterial Protein Toxin Research Cluster, Institute of Molecular Biosciences, Mahidol University, Salaya Campus, Nakornpathom 73170, Thailand; chattipk@gmail.com (C.K.); katzenmeier.ger@mahidol.ac.th (G.K.); 2 Laboratory of Theoretical and Computational Biophysics, Department of Physics, Faculty of Science, Kasetsart University, Bangkok 10900, Thailand; fscicpk@ku.ac.th; 3 Department of Biopharmacy, Faculty of Pharmacy, Silpakorn University, Nakornpathom 73000, Thailand; p.busaba.su@gmail.com; 4 Division of Biochemistry and Biochemical Technology, Department of Chemistry, Faculty of Science, Chiang Mai University, Chiang Mai 50200, Thailand; panchikap@gmail.com; 5 Laboratory of Molecular Biophysics and Chemical Biology, Biophysics Institute for Research and Development (BIRD), Bangkok 10160, Thailand

**Keywords:** *Bordetella pertussis*, CyaA-hemolysin, MD simulations, channel-open lifetime, pore-lining helix, RTX cytolysin, trimeric pore

## Abstract

The *Bordetella pertussis* CyaA-hemolysin (CyaA-Hly) domain was previously demonstrated to be an important determinant for hemolysis against target erythrocytes and ion-channel formation in planar lipid bilayers (PLBs). Here, net-charge variations in the pore-lining helix of thirteen related RTX cytolysins including CyaA-Hly were revealed by amino acid sequence alignments, reflecting their different degrees of hemolytic activity. To analyze possible functional effects of net-charge alterations on hemolytic activity and channel formation of CyaA-Hly, specific mutations were made at Gln^574^ or Glu^581^ in its pore-lining α3 of which both residues are highly conserved Lys in the three highly active RTX cytolysins (i.e., *Escherichia coli* α-hemolysin, *Actinobacillus pleuropneumoniae* toxin, and *Aggregatibacter actinomycetemcomitans* leukotoxin). All six constructed CyaA-Hly mutants that were over-expressed in *E. coli* as 126 kDa His-tagged soluble proteins were successfully purified via immobilized Ni^2+^-affinity chromatography. Both positive-charge substitutions (Q574K, Q574R, E581K, E581R) and negative-charge elimination (E581Q) appeared to increase the kinetics of toxin-induced hemolysis while the substitution with a negatively-charged side-chain (Q574E) completely abolished its hemolytic activity. When incorporated into PLBs under symmetrical conditions (1.0 M KCl, pH 7.4), all five mutant toxins with the increased hemolytic activity produced clearly-resolved single channels with higher open probability and longer lifetime than the wild-type toxin, albeit with a half decrease in their maximum conductance. Molecular dynamics simulations for 50 ns of a trimeric CyaA-Hly pore model comprising three α2-loop-α3 transmembrane hairpins revealed a significant role of the positive charge at both target positions in the structural stability and enlarged diameter of the simulated pore. Altogether, our present data have disclosed functional contributions of positively-charged side-chains substituted at positions Gln^574^ and Glu^581^ in the pore-lining α3 to the enhanced hemolytic activity and ion-channel opening of CyaA-Hly that actually mimics the highly-active RTX (repeat-in-toxin) cytolysins.

## 1. Introduction

*Bordetella pertussis* is a causative agent of human whooping cough (also known as “pertussis”) which has now re-emerged globally as a consequence of pathogen adaptation to vaccination and/or waning protection from acellular pertussis (aP) vaccines [1,2,3]. An improved version of aP vaccines has been made by including additional virulence factors, e.g., adenylate cyclase-hemolysin toxin (CyaA, ~180 kDa), which was previously shown to be an effective protective antigen in mice [4,5]. CyaA is an RTX (Repeat-in-ToXin) cytolysin that facilitates respiratory tract colonization of *B. pertussis* by impairing function of host macrophages [6]. Very recently, we have successfully generated CyaA-specific VH/V_H_H nanobodies that could have a potential for test-driven development of a ready-to-use therapeutic in passive immunization for mitigation of disease severity [7].

**Figure 1:**
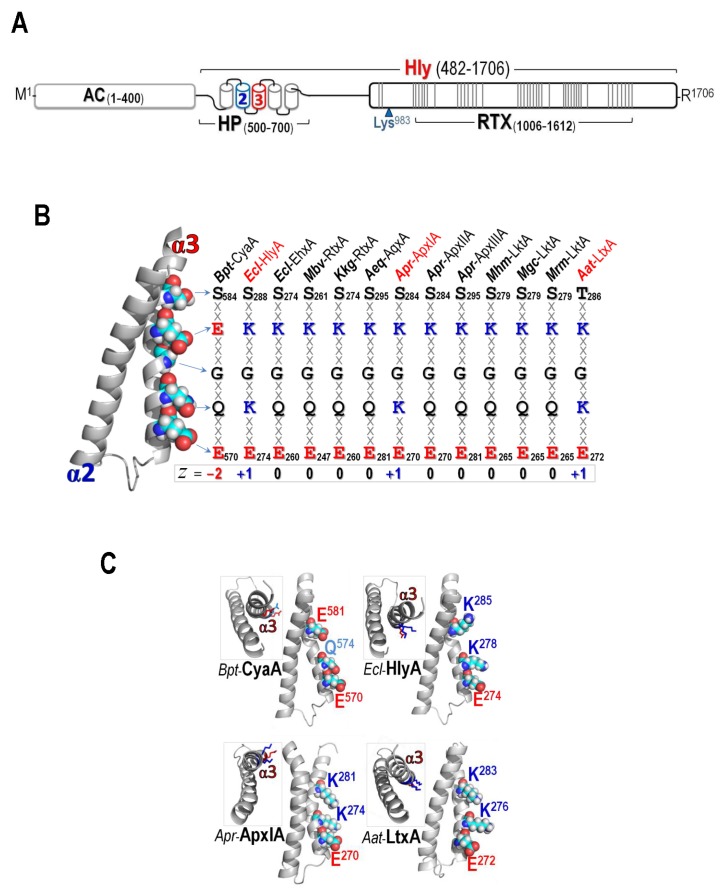
(**A**) (*Above*) Schematic representation of CyaA showing adenylate cyclase (AC) domain and hemolysin (Hly) domain which contains the hydrophobic (HP) region, palmitoylation site (Lys^983^) and the RTX region. Five putative helices in the HP region are indicated by cylinders where α2 and α3, putative membrane-inserting constituents were marked in blue and red, respectively. Each line in the RTX region represents each repeat of (X-U-X-Gly-Gly-X-Gly-X-Asp, X for any amino acid and U for large hydrophobic residues); (**B**) *Left panel*: The homology-based model of the CyaA-Hly α2–α3 hairpin. Side chains at pore-lining region of α3 are shown as van der Waals (vdW) spheres and colored according to atoms: cyan, white, red, and blue for C, H, O, and N, respectively. *Right panel*: Conserved amino acids at pore-lining regions of thirteen related RTX cytolysins, as mentioned earlier in [25]. The side-chains are highlighted in bold letter and the charged residues are colored in red for negative and blue for positive; and (**C**) Side view of the hairpins of CyaA-Hly and three highly-active RTX cytolysins, i.e., HlyA from *E. coli*, ApxIA from *Actinobacillus pleuropneumoniae* and LtxA from *Aggregatibacter actinomycetemcomitans*, showing three key side chains at Glu^570^/Gln^574^/Glu^581^ (for CyaA-Hly) and Glu/Lys/Lys (for HlyA, ApxIA, and LtxA) patches. Inset: Top view of individual hairpins. Amino acids are colored according to their charged/polar properties (red is negatively-charged, blue is positively-charged, and light-blue is N-containing polar uncharged) with H atoms omitted.

The ~180 kDa CyaA toxin is synthesized as a bi-functional polypeptide (1706 residues) of which the *N*-terminal adenylate cyclase domain (AC, 400 residues), complementary to the C-terminal pore-forming/hemolysin domain (Hly, 1306 residues), has made CyaA different from other typical RTX cytolysins [8] (see Figure 1A). In the CyaA-Hly domain, there is a 100 kDa RTX segment containing ~40 repeats of Gly-Asp-rich nonapeptides (X-U-X-Gly-Gly-X-Gly-X-Asp, X for any amino acid and U for large hydrophobic residues) that serves as Ca^2+^-binding sites [9,10,11]. The requirement of Ca^2+^ for either structural stability against proteolytic degradation [11] or proper folding into β-rolls of the RTX sub-domain for accelerating toxin secretion [12] has been evidently demonstrated.

For biological activity, CyaA and/or CyaA-Hly needs a palmitoyl group to be added at Lys^983^ by acyltransferase (CyaC, 21 kDa) [13,14] and is subsequently secreted into the surrounding environment where it selectively binds to the α_M_β_2_ integrin receptor (also known as CD11b/CD18) on human macrophages via the RTX segment [15]. Upon such specific receptor-binding, the ~130 kDa CyaA-Hly domain would mediate translocation of the ~40 kDa catalytic AC domain into the cytosol of target cells causing apoptotic cell death [16]. The attached palmitoyl moiety was suggested to increase membrane affinity of the CyaA toxin needed for target cell attachment by serving either as a mediator of membrane associations or a determinant for receptor-toxin interactions [17]. However, we have recently demonstrated that such palmitoylation at Lys^983^ is not required for the binding of CyaA-Hly to target erythrocyte membranes, but rather needed for stabilizing CyaA-Hly-induced pores/channels [18]. Several studies clearly shown that, without the AC part, the CyaA-Hly domain can independently exert hemolytic activity against sheep erythrocytes that lack the CD11b/CD18 receptor, suggesting an alternative mechanism of target cell recognition via the RTX subdomain [19,20,21,22]. Nevertheless, the exact molecular mechanism of translocation of the AC domain across target cell membranes still needs to be elucidated. Of particular interest, pore formation by the Hly domain is considered as part of the critical step that mediates receptor binding and internalization of the AC domain into the cell cytosol. Better understanding of the behavior of the CyaA/CyaA-Hly-induced pores/channels in model membranes would provide more insight into the structural and biophysical characteristics of the toxin regarding the intoxication process.

Thus far, there is no three-dimensional structure of RTX cytolysins available. We have, therefore, prefigured the *N*-terminal hydrophobic segment (HP, residues 500–700) of CyaA-Hly that could adopt five putative transmembrane helices (i.e., α1_(500–522)_, α2_(529–550)_, α3_(570–93)_, α4_(602–627)_, and α5_(678–698)_) of which α2 and α3 could be the pore-lining constituent participating in hemolytic activity [23]. Our recent studies via single-Ala substitutions in α2 revealed that the Gly^530^_Gly^533^_Gly^537^ cluster, which is arranged as a GXXGXXXG motif, plays a crucial role in CyaA-Hly-induced hemolysis, presumably involved in helix-helix association of the pore-forming helices [24]. We have also demonstrated that two key residues in α3, i.e., Glu^570^ and Glu^581^, are important for toxin activity, conceivably lining the lumen cavity so as to regulate the toxin-induced pore functions [25]. Nonetheless, more structural details of membrane-pore formation by CyaA-Hly still need to be intensively investigated. In the present study, a role of positive charges encompassing the α3-pore-lining region was investigated through net-charged alterations particularly at the two selected positions (i.e., Gln^574^ and Glu^581^). We demonstrate here that replacements with a positively-charged residue at either Gln^574^ or Glu^581^ significantly enhance hemolytic activity of the mutant toxins, consistent with an increase in the lifetime of open channels induced by each mutant toxin in PLBs. Additionally, molecular dynamics (MD) simulations of a CyaA-Hly trimeric pore model (based on our previous quantitative hemolysis assays [26]) in lipid membranes revealed a positive impact of such net-charge alterations, suggesting a significant contribution of positively-charged side-chains at positions Gln^574^ and Glu^581^ in α3 to CyaA-Hly functional activity.

## 2. Results and Discussion

### 2.1. Net-Charge Variations in Pore-Lining Helix of RTX Cytolysins

The HP region of several RTX cytolysins including CyaA has been shown to play a key role in membrane-pore formation [8,23,27]. We previously demonstrated that two putative transmembrane helices (α2 and α3) in the CyaA-Hly domain are crucially involved in hemolytic activity [23,24,25]. Here, as revealed from the Kyte-Doolittle-hydropathy analysis [28], four potential transmembrane segments corresponding to α2–α5 of CyaA-Hly can be identified in the HP region of among thirteen related RTX cytolysins including three highly active members, i.e., *Escherichia coli* α-hemolysin (HlyA), *Actinobacillus pleuropneumoniae* toxin (ApxIA), and *Aggregatibacter actinomycetemcomitans* leukotoxin (LtxA) (see Supplementary Figure S1). This implies that the CyaA-Hly α2/α3 homologues of these related RTX cytolysins could also play an important role in membrane insertion and pore formation. Of particular interest, the α3 homologues that contain highly conserved uncharged polar and charged side-chains (Figure 1B) could conceivably be part of the pore-lining constituent facing the lumen cavity.

Further analysis via multiple alignments of corresponding CyaA-Hly α3 sequences among the thirteen RTX cytolysins shown in Figure 1B revealed variations in their net charge (*z*) that can be categorized into three groups: (*i*) a negatively-charged patch (*z* = −2) of CyaA-Hly, containing Glu^570^/Gln^574^/Glu^581^; (*ii*) a positively-charged patch (*z* = +1) of HlyA, ApxIA, and LtxA, containing Glu/Lys/Lys; and (*iii*) a neutral patch (*z* = 0) of nine other RTX cytolysins, containing Glu/Gln/Lys. It is important to note that the group with *z* = +1 (i.e., *Ecl*-HlyA, *Apr*-ApxIA, and *Aat*-LtxA) exhibited a very strong hemolytic activity while the group with *z* = −2 (i.e., CyaA-Hly) displayed a relatively weak activity [6,29,30,31]. Thus, such surface net-charge variations in the pore-lining helix of these RTX cytolysins could reflect their different degrees of hemolytic/pore-forming activities.

### 2.2. Effects of Net-Charge Alterations at Gln^574^ and Glu^581^ on CyaA-Hly Hemolysis

**Figure 2:**
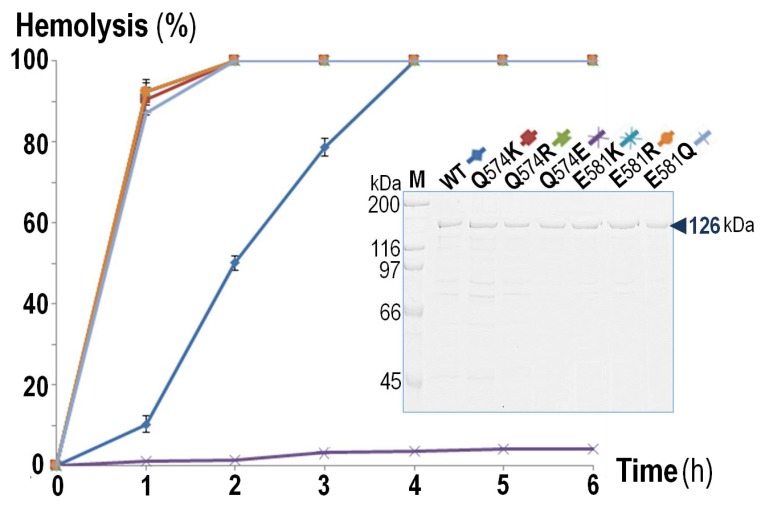
Time-course of hemolytic activity against sheep erythrocytes of individual purified toxins (10 μg/mL) as indicated. The control for 100% hemolysis is susceptible sheep erythrocytes with respect to a complete lysis when treated with 0.1% Triton X-100. Subset: SDS-PAGE (10% gel) of the Ni^2+^-NTA purified CyaA-Hly toxins, both wild-type (WT) and mutants (Q574K, Q574R, Q574E, E581K, E581R, and E581Q). Bands of CyaA-Hly toxins 126 kDa in length are marked by an arrow.

To test whether a positive net-charge covering the pore-lining region is essentially associated with a strong hemolytic activity of RTX cytolysins, we carried out net-charge alterations in the negatively-charged patch (*z* = −2) of CyaA-Hly as a study model. It is worth mentioning that the negatively-charged Glu^570^ which is one of the patch constituents (Glu^570^/Gln^574^/Glu^581^) in CyaA-Hly α3 and is highly conserved throughout the RTX cytolysin family, was previously demonstrated to be important for hemolytic activity of CyaA-Hly [25]. We, therefore, focused on only two other positions, i.e., Gln^574^ and Glu^581^, of which both positions in all the three highly-active RTX cytolysins (i.e., *Ecl*-HlyA, *Apr*-ApxIA, and *Aat*-LtxA) have a preference for positively-charged Lys (see Figure 1B,C). Although, to our knowledge, these two highly-conserved Lys side-chains have not yet been characterized for their functional significance, they might possibly contribute to the high hemolytic activity of *Ecl*-HlyA, *Apr*-ApxIA, and *Aat*-LtxA. Herein, a total of six single-substituted mutants (Q574K, Q574R, Q574E, E581K, E581R, and E581Q) at both Gln^574^ and Glu^581^ were constructed corresponding to either charge alteration or elimination. Upon induction with isopropyl-β-d-thiogalactopyranoside (IPTG), all verified CyaA-Hly mutant clones were able to express 6 × His-tagged soluble proteins (CyaA-Hly/H_6_) of 126 kDa at high levels comparable to the wild-type clone (see Supplementary Figure S2). Further analysis by Western blotting confirmed that all the CyaA-Hly/H_6_ mutant toxins were recognized by both anti-His and anti-RTX antisera, verifying the presence of a 6 × His-affinity tag as well as its RTX identity. Subsequently, the wild-type and mutant toxins were successfully purified using the same immobilized Ni^2+^-affinity chromatographic conditions, suggesting that they all preserved their Ni^2+^-NTA binding characteristics. As analyzed by SDS-PAGE, the 126 kDa protein band with >90% purity of all the CyaA-Hly/H_6_ mutants was efficiently obtained as good as the wild-type toxin via a stepwise elution with 75 mM imidazole (Figure 2 subset).

To further determine an effect of these net-charge alterations on CyaA-Hly hemolysis, we tested all individual mutant toxins for their relative hemolytic activity against susceptible sheep erythrocytes. The hemolysis data shown in Figure 2 reveal that the purified mutant toxins obtained via positive-charge substitutions (Q574K, Q574R, E581K, E581R) or the negative-charge elimination (E581Q) could exhibit a significantly enhanced activity as they cause complete hemolysis against sheep erythrocytes within 2 h, much faster than the wild-type toxin, which required up to 4 h. However, the negative-charge substitution at Gln^574^ (Q574E) completely eliminated toxin hemolytic activity. These results supported the preference of a positive charge at either position (Gln^574^ or Glu^581^) facing the pore interior of which its net charge has changed from −2 to −1 for Q574K and Q574R mutants and from −2 to 0 for E581K and E581R mutants. Moreover, the non-preference of the negative charge was confirmed by the enhanced activity of the E581Q mutant (*z* decreased from −2 to −1) and the impaired activity of the Q574E mutant (*z* increased from −2 to −3). Loss in hemolytic activity of the Q574E mutant might be due to a local structure distortion caused by the introduced negatively-charged Glu that faces the same side and is close enough to generate charge-charge repulsion with the key functional residue-Glu^570^ (see Figure 1C). Accordingly, our results demonstrated that specific mutations at Gln^574^ or Glu^581^ in CyaA-Hly α3 for resembling the positively-charged patch (*z* = +1) of the highly-active RTX cytolysins (i.e., *Ecl*-HlyA, *Apr*-ApxIA and *Aat*-LtxA) could improve lytic pore-forming efficiency of CyaA-Hly toward sheep erythrocytes. Thus, these results support an important contribution of a positive net-charge covering the pore-lining region of CyaA-Hly α3 to an enhancing effect on toxin hemolytic activity.

### 2.3. Ion-Channel Characteristics Formed by CyaA-Hly Mutant Toxins with Enhanced Hemolytic Activity

**Figure 3:**
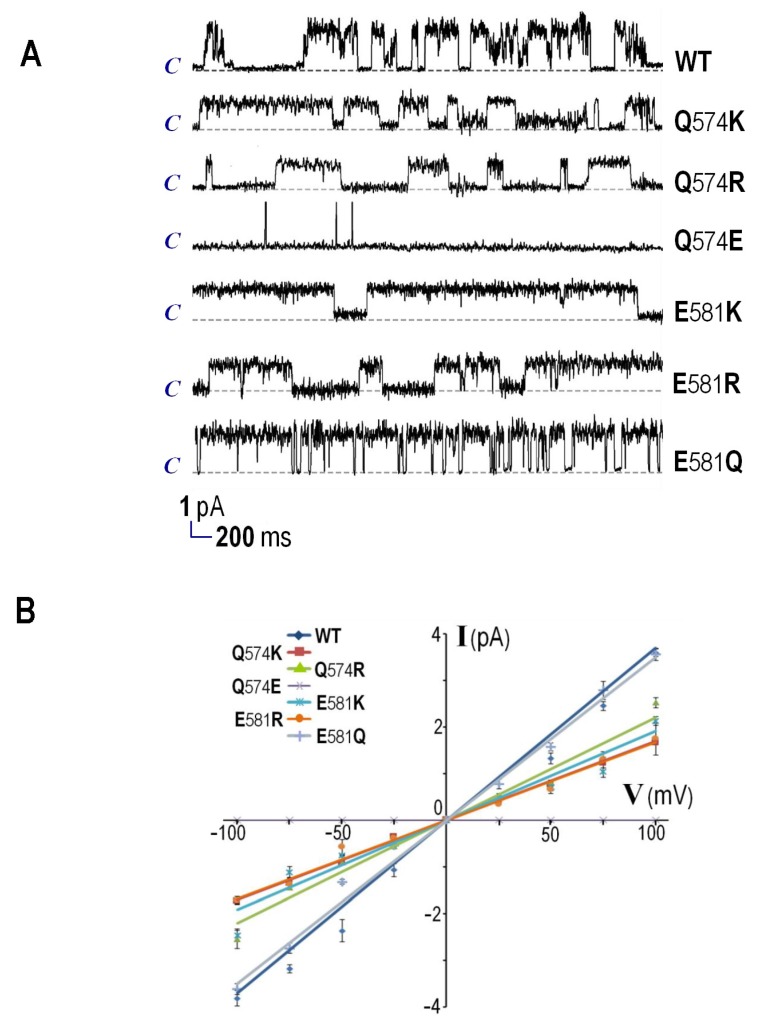
Ion-channel properties of CyaA-Hly wild-type (WT) and its mutant toxins (1 μg/mL). (**A**) Representative current traces were recorded at 100 mV holding potential. The closed stage level of channels is denoted by the letter *c*. Vertical and horizontal bars indicate measured current-time scales, respectively; and (**B**) current-voltage relations of all single channels formed by WT and its mutant toxins were recorded under symmetric conditions.

Experiments were further conducted to assess channel-forming properties of the purified CyaA-Hly/H_6_ mutants in comparison with that of the wild-type toxin. By incorporation into PLBs under symmetrical ionic conditions (at 1 M KCl, 10 mM HEPES, pH 7.4), the wild-type toxin could cause ion channels to open with a maximum conductance (*g*_K_) of 36.4 ± 1.7 pS, as shown by representative current traces (Figure 3A). However, the positive-charge substituted mutants with enhanced hemolytic activity, i.e., Q574K, Q574R, E581K, and E581R, could induce the channels to open with approximately a half decrease in *g*_K_ (17.1 ± 0.2, 22.0 ± 0.3, 19.3 ± 0.2, and 16.4 ± 0.6 pS, respectively), except for the E581Q mutant of which *g*_K_ remained relatively unchanged (34.7 ± 0.4 pS) (see Figure 3B and Table 1). It was also found that the hemolytically-inactive Q574E mutant was unable to produce channel conductance in PLBs, confirming the non-preference of the negatively-charged side-chain at this position.

Remarkably, the channels induced by these positive-charge substituted mutants: Q574K, Q574R, E581K, and E581R, appeared to have significant longer open lifetimes (i.e., 1620, 2980, 1920 and 2500 ms, respectively) than that induced by wild-type toxin (~235 ms) while the E581Q mutant’s channel exhibited a slightly shorter lifetime (~150 ms) (see Table 1). In addition, all the active mutants showed a marked increase in the open channel probability (0.78, 0.53, 0.71, 0.82, and 0.76 for Q574K, Q574R, E581K, E581R, and E581Q, respectively) compared to the wild-type toxin (0.29). It is possible that the channel interior with less negative charge could prolong open-channel lifetimes and increase open-channel probability, conceivably accounting for their enhanced hemolytic activity. As revealed in certain ion channels and transporters, the preference of charged residues at particular locations inside the pores can affect their structural arrangement through interactions between neighboring oppositely-charged residues [32,33,34]. However, all four hemolytically-active mutants substituted with a positive charge (i.e., Q574K, Q574R, E581K, and E581R) appeared to produce single-channels with about a half decrease in maximum conductance compared to the wild-type and E581Q mutant toxins as demonstrated above (see Figure 3). Therefore, an introduced positively-charged side-chain at these two positions could perhaps disturb cation-conducting ability of CyaA-Hly channels, implying the disparate manners of CyaA-Hly in regulating between its hemolytic pore formation and ion-conduction.

### 2.4. Trimeric Pore Models and Effects of Net-Charge Alterations on Their Structural Dynamics

To gain more insights into the architecture of the CyaA-Hly pore/channel, we constructed a plausible 3D-modeled pore by docking three copies of the α2-loop-α3 hairpin, based on osmotic protection experiments, which estimated the CyaA-induced pore diameter to be approximately 6–8 Å [35], and our previous quantitative hemolysis assays, which revealed that the toxin could cooperatively form a trimeric pore with a Hill coefficient of ~3 [26]. This similar finding could be observed in some other bacterial protein toxins from entirely different family, for instance, *Bacillus thuringiensis* Cry4Ba insecticidal protein, which was also demonstrated to form a trimeric pore structure [36,37]. These two pore-forming toxins potentially share a similar pore structure and mechanism of pore formation, although they have no sequence similarities outside of their pore-lining constituents. Thus, nature seems to have developed an analogous design for the two toxin-induced pores/channels by different evolutionary pathways.

**Figure 4:**
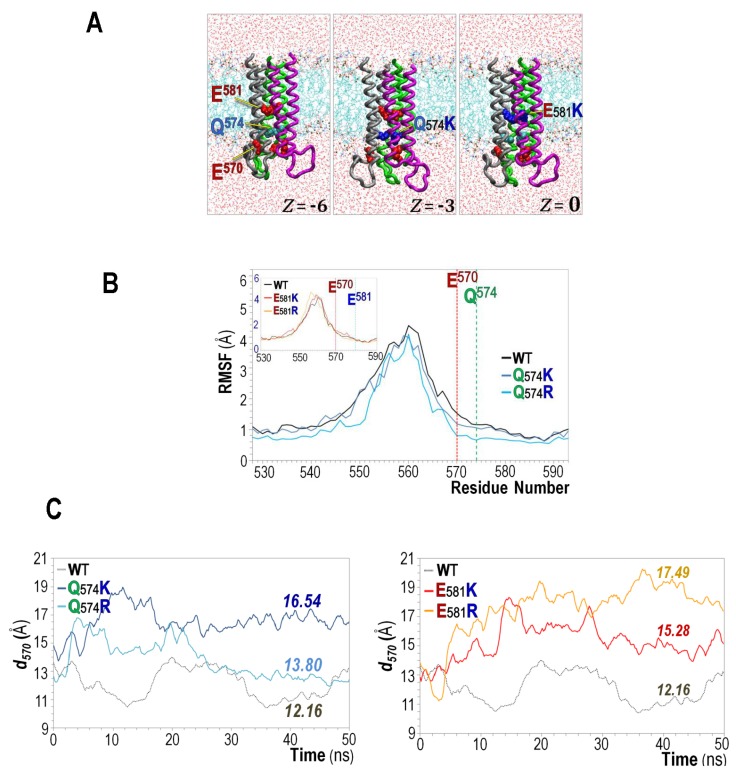
(**A**) MD simulations (50 ns) of trimeric CyaA-Hly channel models in membrane of the wild-type (WT, left) and two representative mutants; Q574K (middle) and E581K (right). Total net charges (*z*) inside the pores are shown; (**B**) C_α_-RMSF profiles (Arg^528^–Ala^593^) obtained from MD trajectories of 50-ns simulations of individual CyaA-Hly pore structures; and (**C**) the average separation distance (C_α_ to C_α_) between three Glu^570^ residues on the trimeric CyaA-Hly pore models versus time during 50-ns MD simulations. *Left* and *right panels* represent the systems of Gln^574^ and Glu^581^ mutants compared to the wild-type, respectively.

A possible role of the positive charge substituted at either critical position (Gln^574^ or Glu^581^) conceivably located at the CyaA-Hly pore interior was subsequently characterized via 50-ns MD simulations of four individual trimeric pore-mutant models with positive-charge substitutions, Q574K, Q574R, E581K, and E581R (see Figure 4A). As further illustrated in Figure 4B, replacement with a positive charge (either Lys or Arg) at Gln^570^ can reduce structural fluctuation of both Q574K- and Q574R-simulated pores shown by their decreased RMSF values, especially around the intracellular pore mouth at Glu^570^. It is conceivable that Glu^570^ and Lys/Arg-substituted side-chain at Gln^574^ which are oriented closely facing the pore lumen could perhaps create electrostatic interactions to stabilize the pore mouth. Nevertheless, the effect on RMSF was not significant when Lys/Arg was introduced at Glu^581^ located rather far from Glu^570^ (see Figure 4B inset). Interestingly, positive-charge substitutions at either Gln^574^ or Glu^581^ were both found to increase pore diameter around the intracellular entry, as presented by the averaged separation distance (*d*) of Glu^570^, which is ~12.2 Å for the wild-type, and increases to about 16.5, 13.8, 15.3, and 17.5 Å for Q574K, Q574R, E581K, and E581R mutants, respectively (see Figure 4C). The increased stability and pore size of the Lys/Arg-substituted mutant pores observed herein were consistent with their prolonged open-channel lifetimes and increased open-channel probability in the PLBs as demonstrated above (see Table 1).

Taken as a whole, we have clearly demonstrated that CyaA-Hly can replicate other highly-active RTX cytolysins (e.g., *Ecl*-HlyA, *Apr*-ApxIA, and *Aat*-LtxA), and becomes much more hemolytically active by introducing a positively-charged side-chain at Gln^574^ or Glu^581^ (or by removing a negatively-charged side-chain at Glu^581^) in the pore-lining α3. The increase in the hemolytic activity of the CyaA-Hly mutants, i.e., Q574K, Q574R, E581K, E581R, and E581Q, could perhaps come from the prolonging of the pore in its open state as revealed by PLB results. Additionally, this is probably due to the larger pore diameter and more structural stability of the mutant pores as indicated in the MD simulations. Such a stable-large pore of the mutants would permeable to all ions, promoting a faster hemolysis of target erythrocytes. Moreover, introducing a negatively charged side-chain at Gln^574^ could give the opposite effect as the pores destabilized and became hemolytically inactive. However, in PLBs, only half conductance was observed for mutants with a positive-charge substitution, albeit being very hemolytically active against sheep erythrocytes. The explanation of this is that the introduced positive charge at whichever side-chain (i.e., Gln^574^ or Glu^581^) could possibly do two things, (i) it would create a positive electrostatic energy barrier that prevent cations to flow through the pore (as CyaA-induced channels were shown to be cation-selective [29]) and hence the reduction in conductance; and (ii) it, not only at Glu^581^ but also at Gln^574^, would stabilize the pore structure by balancing the strong electrostatic repulsions between Glu^570^ and Glu^581^ in the pore and hence the overall increase in hemolytic activity of the positive-charge substituted toxins. Thus, these findings provide insights to better understanding about membrane-pore formation of the RTX cytolysins that net-charge variations at the pore-lining region could be a factor accounting for their differences in pore-forming efficiency.

## 3. Materials and Methods

### 3.1. Hydropathy Analysis and Protein Multiple Sequence Alignments

Hydropathy plots of the HP regions from thirteen related RTX cytolysins, i.e., CyaA from *B. pertussis* (*Bpt*-CyaA: CAE41066), HlyA and EhxA from *E. coli* (*Ecl*-HlyA: ABE10329 and Ecl-EhxA: BAA31774), RtxA from *Moraxella bovis* (*Mbv*-RtxA: AKK84651), and *Kingella kingae* (*Kkg*-RtxA: ABK58601), AqxA from *Actinobacillus equuli* (*Aeq*-AqxA: AMM45569); ApxIA, ApxIIA and ApxIIIA from *A. pleuropneumoniae* (*Apr*-ApxIA: AAL55666, *Apr*-ApxIIA: AAU84700 and *Apr*-ApxIIIA: CAA48711), LktA from *Manheimia haemolytica* (*Mhm*-LktA: AAL13281), *M. glucosida* (*Mgc*-LktA: AAG40306), and *M. ruminalis* (*Mrm*-LktA: AAR09165) and LtxA from *Aggregatibacter actinomycetemcomitans* (*Aat*-LtxA: CAA34731), were performed as described by Kyte and Doolittle [28] with a window size of 19 residues. Multiple alignments of protein sequences were accomplished using Clustal server (Clustal W 2.0, 2007, Oxford University Press, Oxford, UK) [38].

### 3.2. Homology-Based Modeling of Hairpin Structures

Atom coordinates of α2-loop-α3 hairpin were extracted from the homology-based model of the CyaA-HP region (α1–α5), which was previously constructed [26]. The extracted CyaA-hairpin was subsequently used as a template for modeling its corresponding hairpin structures of relative RTX cytolysins via SWISS-MODEL server (2003, Oxford University Press, Oxford, UK) [39]. Modeled hairpin structures were energy-minimized using NOMAD-Ref server (2006, Oxford University Press, Oxford, UK) [40].

### 3.3. Construction of Mutant Plasmids

The pCyaAC-PF/H_6_ plasmid encoding 126 kDa 6 × His-tagged CyaA-Hly [26] was used as a template. Complementary pairs of mutagenic primers were designed based on the CyaA-Hly gene sequence (see Supplementary Table S1). All mutant plasmids were generated by polymerase chain reaction (PCR)-based mutagenesis using high-fidelity Phusion DNA polymerase (Finnzymes, Vantaa, Finland), following the QuickChange^TM^ mutagenesis procedure (Agilent Technologies, Santa Clara, CA, USA). Mutant plasmids were first identified by restriction endonuclease digestion and subsequently verified by DNA sequencing.

### 3.4. Bacterial Culture and Toxin Expression

*E. coli* strain BL21(DE3)pLysS was individually transformed with each mutant plasmids and cultured at 30 °C in Terrific broth containing ampicillin (100 μg/mL) and chloramphenicol (34 μg/mL) as previously described [26]. When the cultures reached 0.5 OD_600_, protein expression was induced by IPTG (isopropyl-β-d-thiogalactopyranoside) at a final concentration of 0.1 mM and then incubated for additional 4 hr.

### 3.5. Toxin Purification via Immobilized Metal Affinity Chromatography

Cultured cells were harvested (6000× *g*, 4 °C, 10 min), re-suspended in 50 mM Tris-HCl (pH 7.4) supplemented with 5 mM CaCl_2_ and 1 mM protease inhibitors (phenylmethylsulfonylfluoride and 1,10-phenanthroline), and subsequently disrupted in a French Pressure Cell (10,000 psi). After centrifugation (13,000× *g*, 4 °C, 15 min), His-tagged soluble toxins in lysate supernatant were purified using the immobilized metal affinity column (Ni^2+^-NTA) as previously described [26]. Protein concentrations were quantified using Bradford microassay (Bio-RAD, Hercules, CA, USA) and then analysed by SDS–PAGE.

### 3.6. Toxin Verification via Western Blot Analysis

Protein toxins from SDS-PAGE were electroblotted onto a nitrocellulose membrane and then blocked with 5% non-fat milk in PBS (120 mM NaCl, 16 mM Na_2_HPO_4_, 4 mM NaH_2_PO_4_, pH 7.4) prior to probing with anti-RTX antibodies (1:40,000 dilution) which was raised against the 100 kDa CyaA-RTX subdomain fragment [11]. Immune-complexes were detected with anti-rabbit IgG 2° antibodies conjugated with alkaline phosphatase (AP, 1:7000 dilution; Pierce, Rockford, IL, USA) and then immuno-reactive bands were visualized by color development with 5-bromo-4-chloro-3-indolyl phosphate/nitroblue tetrazolium substrates (KPL, Gaitherburg, MD, USA). The presence of the 6 × His tag was verified by probing with AP-conjugated anti-His (C-term) antibodies (1:2500 dilution; Invitrogen, Waltham, MA, USA) and BCIP/NBT color detection.

### 3.7. Determination of Hemolytic Activity of Mutant Toxins

The purified toxin (~10 μg/mL) was assayed with sheep erythrocyte suspension (5 × 10^8^ cells/mL in 150 mM NaCl, 2 mM CaCl_2_, 20 mM Tris-HCl, pH 7.4) at 37 °C for 6 h. Non-lysed cells and debris were removed by centrifugation at (12,000× *g*, 2 min), and the supernatant was transferred to a 96-well microtiter plate for determining the released hemoglobin by measuring absorbance at 540 nm (OD_540_). Percent hemolysis for each toxin sample was calculated by [(OD_540_ sample − OD_540_ negative control)/(OD_540_ of 100% hemolysis − OD_540_ negative control)] × 100. An equal amount of erythrocytes lysed with 0.1% Triton X-100 was defined as 100% hemolysis. All samples were tested in triplicate for three independent experiments as previously described [21].

### 3.8. Single-Channel Analysis of Mutant Toxins via Planar Lipid Bilayers (PLBs)

PLBs were formed by painting method 20 mg/mL of 1,2-diphytanoyl-*sn*-glycero-3- phosphocholine (DiPhyPC, Avanti Polar Lipids, Alabaster, AL, USA) on a 250-μm aperture in a 1 mL-Delrin cup (Warner Instruments, Hamden, CT, USA) as previously described [26]. Toxin samples (1 μg/mL; prepared in 20 mM HEPES, pH 7.4, 5 mM CaCl_2_) were added into both *cis* and *trans* compartments containing recording buffer (1 M KCl, 10 mM HEPES, pH 7.4). Single-channel currents were recorded with Geneclamp-500 amplifier (Axon Instruments, Sunnyvale, CA, USA) and signals (filtered at 10 kHz) were digitized with PCI-6221 analog-to-digital converter (National Instruments, Austin, TX, USA) using LabVIEW 7.1 software at a 50 kHz sampling frequency. Channel conductances and lifetimes were determined from the observed current steps (~100 open channels) at 100 mV applied voltage.

### 3.9. Trimeric Docking and Molecular Dynamics (MD) Simulations of a Modeled Pore

A trimeric pore model of CyaA-Hly was constructed by docking three copies of the α2-loop-α3 hairpin using ZDOCK server (ZDOCK 3.0.2, 2014, Oxford University Press, Oxford, UK) [41] as previously described [24]. The model was inserted into a DMPC (1, 2 dimyristoyl-*sn*-glycero-3-phosphocholine) bilayer using CHARMM GUI server (2014, John Wiley & Sons Inc., New York, NY, USA) [42] and then water and ions were added to obtain a system of CyaA-Hly trimeric pore at 150 mM KCl using VMD program [43]. The final system has 40,301 atoms consisting of the trimeric pore, 120 DMPC molecules, 8748 water molecules, 27 K^+^ ions and 27 Cl^−^ ions (at 150 mM KCl) was energetically minimized, equilibrated, and finally ran for 50 ns at temperature 300 K and pressure 1 atm using the NAMD program [44] and CHARMM force-fields [45] on a computer cluster with 16 CPU-cores (2.3 GHz AMD Opteron) at 2 ns per day. Full electrostatic calculation with multiple-time stepping was used with periodic boundary conditions and 2 fs integration time. All other MD simulation parameters were set as described in [46]. The dynamical and structural properties of the trimeric pore model were analyzed from the MD trajectories using VMD program. Root mean square fluctuation (RMSF) was calculated from RMSF = $\sqrt{\frac{1}{T}\sum_{t=1}^{T}{\left[x_{i}\left(t\right)-{\tilde{x}}_{i}\right]}^{2}}$, where $x_{i}\left(t\right)$ and ${\tilde{x}}_{i}$ are the coordinates of C_α_ atom of residue i at time-step t, and their average over *T* time-steps, respectively.

## Figures and Tables

**Table 1:** Channel characteristics of CyaA-Hly wild type and mutants.

Toxins ^a^	*z* ^b^	Hemolytic Activity ^c^	*g*_K_ (pS) ^d^	Open Lifetime (ms) ^e^	Open Probability ^f^
WT	−6	10.1 ± 1.1%	36.1 ± 1.7	236 ± 1	0.29
Q574K	−3	35.9 ± 1.0%	17.1 ± 0.2	1620 ± 12	0.78
Q574R	−3	36.6 ± 0.7%	22.0 ± 0.3	2980 ± 20	0.53
Q574E	−9	1.6 ± 0.1%	- ^g^	- ^g^	- ^g^
E581K	0	35.1 ± 1.1%	19.3 ± 0.2	1920 ± 16	0.71
E581R	0	30.0 ± 1.0%	16.4 ± 0.6	2500 ± 14	0.82
E581Q	−3	32.3 ± 1.0%	34.7 ± 0.4	147 ± 2	0.76

^a^ Ni^2+^-NTA purified toxins; ^b^ Surface charge of interior pore in trimeric model; ^c^ % Hemolytic activities of CyaA-Hly toxins (1 μg/mL) against sheep erythrocytes at 6 h; ^d^ The single channel conductance of 1 μg/mL toxins recorded in 1 M KCl solution (pH 7.4), and presented as mean ± SEM from three independent experiments; ^e^ The open channel lifetime was calculated from ~100 open events in 1 M KCl solution (pH 7.4) at 100 mV holding potential from equation; N(*t*)/N(0) = exp(−*t*/τ); ^f^ Open probability was calculated from ~100 open events in 1 M KCl solution (pH 7.4) at 100 mV holding potential from equation *P_open_* = *t_open_*/(*t_open_* + *t_closed_*); ^g^ The event cannot be observed.

## Supplementary Materials

The following are available online at www.mdpi.com/2072-6651/9/3/109/s1, Table S1: Complementary primers used in mutagenesis, Figure S1: Hydropathy profile of the hydrophobic (HP) region from CyaA-Hly (blue line) compared with three highly-active RTX cytolysins i.e., HlyA, ApxIA and LtxA (green lines), Figure S2: SDS-PAGE (12% gel) of the soluble lysates extracted from *E. coli* harboring different plasmids; *pET-17b* vector, pCyaAC-PF/H_6_ (Wt) and pCyaAC-PF/H_6_ with mutations (Q574K, Q574R, Q574E, E581K, E581R and E581Q).

## Abbreviations

The following abbreviations are used in this manuscript:

CyaA	adenylate cyclase-hemolysin toxin
DiPhyPC	1,2-diphytanoyl-*sn*-glycero-3-phosphocholine
DMPC	1, 2 dimyristoyl-*sn*-glycero-3-phosphocholine
MD	molecular dynamics
Ni^2+^-NTA	nickel-nitrilotriacetic acid
PLBs	planar lipid bilayers
RTX	Repeat-in-Toxin
VH/V_H_H	variable heavy chain domain
