# Supplementary material for: Functional Contributions of Positive Charges in the Pore-Lining Helix 3 of the Bordetella pertussis CyaA-Hemolysin to Hemolytic Activity and Ion-Channel Opening
Source: Toxins (Basel). 2017 Mar 16;9(3):109. doi: 10.3390/toxins9030109 (PMC5371864; doi:10.3390/toxins9030109)
Supplement: Supplementary file 1 [file toxins-09-00109-s001.pdf]

Supplementary Materials: Functional Contributions of Positive Charges in the Pore-Lining Helix 3 of the *Bordetella pertussis* CyaA-Hemolysin to Hemolytic Activity and Ion-Channel Opening

Chattip Kurehong, Chalermpol Kanchanawarin, Busaba Powthongchin, Panchika Prangkio, Gerd Katzenmeier and Chanan Angsuthanasombat

Table S1. Complementary primers used in mutagenesis.

Primer [a]	Sequences [b]	Restriction site [c]
Q574K-f<br>Q574K-r	5′-GCGCTGAAATTGACAGGTGGAACG-3′<br>3′-GGCTCTAGCGCGACTTTAACTGTCC-5′	*Pst*I
Q574R-f<br>Q574R-r	5′-GCGCTGCGCTTGACAGGTGGAACG-3′<br>3′-GGCTCTAGCGCGACGCGAACTGTCC-5′	*Pst*I
Q574E-f<br>Q574E-r	5′-GCGCTGGAATTGACAGGTGGAACG-3′<br>3′-GGCTCTAGCGCGACCTTAACTGTCC-5′	*Pst*I
E581K-f<br>E581K-r	5′-ACGGTCAAACTGGCTTCTTCCATCG-3′<br>3′-TGTCCACCTTGCCAGTTTGACCGA-5′	*Alu*I
E581R-f<br>E581R-r	5′-ACGGTCCGCCTGGCTTCTTCCATCG-3′<br>3′-TGTCCACCTTGCCAGGCGGACCGA-5′	*Alu*I
E581Q-f<br>E581Q-r	5′-ACGGTCCAACTGGCTTCTTCCATCG-3′<br>3′-TGTCCACCTTGCCAGGTTGACCGA-5′	*Alu*I

[a] f and r represent forward and reverse primers, respectively. [b] Underlined bases represent the recognition sites introduced for restriction enzyme analysis. Bold letters indicate the substituted nucleotide residues. The mutated residues in introduced restriction sites generate silent mutations. [c] Deleted recognition sites.

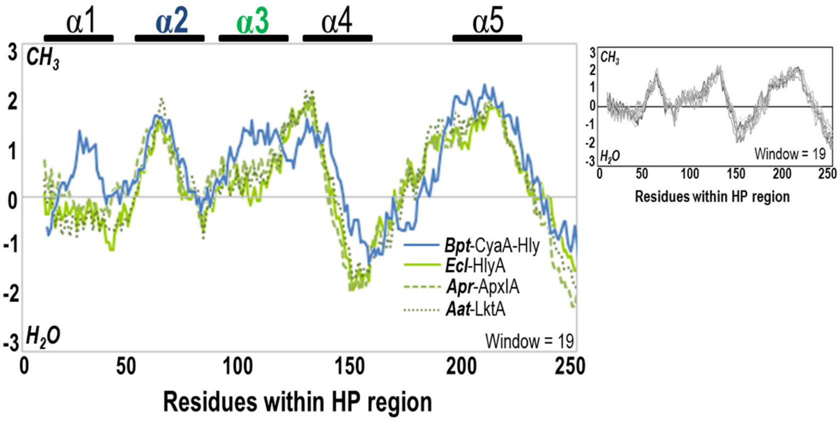

Figure S1. Hydropathy profile of the hydrophobic (HP) region from CyaA-Hly (blue line) compared with three highly-active RTX cytolysins i.e. HlyA, ApxIA and LtxA (green lines). The hydrophobicity scale was plotted versus amino acid sequences (~250 residues) covering the hydrophobic regions with a window size of 19 using Kyte-Doolittle hydropathy index. Five putative helices in the HP region of CyaA-Hly were indicated. Inset: the hydropathy profile of the HP region from all twelve related RTX cytolysins as mentioned earlier in [25].

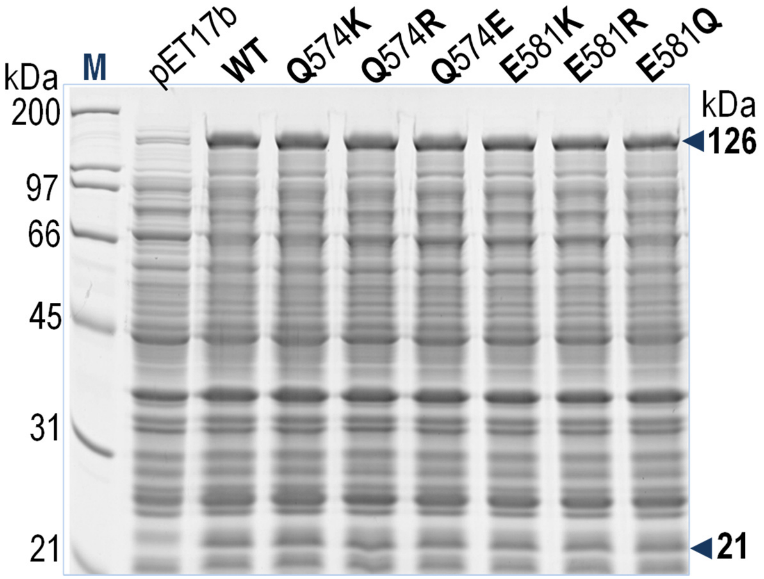

Figure S2. SDS-PAGE (12% gel) of the soluble lysates extracted from *E. coli* harboring different plasmids; pET-17b vector, pCyaAC-PF/H_6 (Wt) and pCyaAC-PF/H_6 with mutations (Q574K, Q574R, Q574E, E581K, E581R and E581Q). Co-expressed with 126 kDa CyaA-Hly is the 21 kDa CyaC acetyltransferase marked by arrow. M is protein standard marker.
